# Cancer incidence in eastern Morocco: cancer patterns and incidence trends, 2005–2012

**DOI:** 10.1186/s12885-017-3597-6

**Published:** 2017-08-29

**Authors:** Manal Elidrissi Errahhali, Mounia Elidrissi Errahhali, Meryem Ouarzane, Redouane Boulouiz, Mohammed Bellaoui

**Affiliations:** Genetics Unit, Faculty of Medicine and Pharmacy of Oujda, University Mohammed Premier, Oujda, Morocco

**Keywords:** Cancer patterns, Incidence, Time trends, Cancer control, Morocco

## Abstract

**Background:**

Cancer is one of the major health problems worldwide. In this article, we present for the first time the cancer incidence trends, the distribution and the socioeconomic profile of incident cancer cases in Eastern Morocco over a period of eight years.

**Methods:**

Retrospective descriptive study of patients diagnosed with cancer at the Hassan II Regional Oncology Center (ROC) since it was created in October 2005 until December 2012. During the study period, the ROC was the only hospital specialized in cancer care in Eastern Morocco.

**Results:**

A total of 7872 incident cases of cancer were registered in Eastern Morocco. Among these incident cases 5220 cases were women and 2652 were men, with a female to male ratio of 1.97. The mean age at diagnosis was 58 years for males and 52 for females and 94% of the patients aged over 30 years. For both sexes combined and for all cancer sites, breast cancer was the commonest followed by cervix uteri, colon-rectum, lung, nasopharynx, and stomach cancers. The most common cancer in women was breast cancer, followed respectively by cervix uteri cancer, colon-rectum cancer, ovary cancer, and stomach cancer. In men, the lung cancer ranked first, followed respectively by colon-rectum cancer, nasopharynx cancer, prostate cancer, and stomach cancer. For most cancers, crude incidence rates (CR) have increased significantly. The CR for all cancers combined has increased from 56.6 to 80.3 per 100,000 females and from 32.3 to 42.6 per 100,000 males during the study period. Patients profile analysis showed that 79% of cancer patients were from urban areas, 83% were unemployed and 85% had no health insurance.

**Conclusions:**

The distribution of cancers in Eastern Morocco is different from those observed in other regions of Morocco. Unlike most countries, women were much more affected with cancer than men in Eastern Morocco. More importantly, the rates of many cancers are rising. Therefore, our data justify the need to develop effective programs for cancer control and prevention in Eastern Morocco. A better access to cancer care should be a priority of the health policies, given that the majority of cancer patients in Eastern Morocco are unemployed, and do not have medical care coverage.

## Background

According to WHO estimates, cancer causes more deaths than all cardiovascular diseases [1]. Therefore, cancer has become a major public health problem worldwide [2, 3]. The most common tumors worldwide were lung cancer with 1.8 million cases (13.0% of total), breast cancer with 1.7 million cases (11.9% of total) and the colorectal cancer with 1.4 million cases (9.7% of total). Similarly, cancers that cause the highest rate of deaths are cancers of the lung (1.6 million deaths, 19.4% of total), liver (0.8 million deaths, 9.1% of total) and stomach (0.7 million deaths, or 8.8% of total) [1]. In 2012, the overall cancer burden reached 14.1 million new cases compared to 12.7 million in 2008 [4, 5]. Similarly, 8.2 million deaths have been attributed to cancer during 2012 (13% of all deaths worldwide) compared to 7.6 million deaths in 2008 [4, 5].

In 2012, more than half of all cancer cases (56.8%) and deaths caused by cancer (64.9%) were recorded in the least developed regions of the world [1]. For example in Africa, there were 846,961 cases of cancer and 591,161 cancer deaths in 2012 [1]. Accordingly, epidemiologic studies predict 1.2 million new cancer cases in Africa with more than 970,000 deaths by 2030 if adequate control and prevention measures are not taken promptly [1, 6].

In Morocco, cancer is a major health problem and it is the second leading cause of mortality after cardiovascular diseases with 10.7% of all deaths [7]. However, there are only two population-based cancer registries in Morocco at the present time. The Casablanca registry was founded in March 2003 and the Rabat registry was created in January 2005. These registries have provided important information on cancer patterns in western Morocco [8–12]. In Eastern Morocco, nothing is known about cancer incidence and therefore, in this study, we report for the first time the cancer incidence trends, the distribution and the socioeconomic profile of incident cancer cases in Eastern Morocco over a period of eight years between 2005 and 2012.

## Methods

### Setting

Eastern Morocco is located in the north east of the kingdom of Morocco. According to the High Commission for Planning (HCP), Eastern Morocco had a population of over 2 million in 2013, which is equivalent to 6.2% of the total population of the Kingdom. The population is mainly urban (67% vs. 33% rural) and young, nearly 6 out of 10 people are under 30 years [13–16].

Our retrospective study was based on all incident cases that were registered at the Hassan II Regional Oncology Center, since it was created in October 2005 until December 2012. During the study period, the Hassan II Regional Oncology Center (ROC) was the only health care facility for management of all solid cancer cases among adult patients in Eastern Morocco.While during this period, childhood cancer cases were treated in other health care facilities in the capital of Morocco (Rabat). However, hematological malignancies (HM) were managed in different centres: Al-Farabi Regional Hospital, Boussif Diagnostic Center, ROC, and some health care facilities in Rabat [13, 15].

### Data collection and cancer classification

The data were collected from patient medical records, pathology records and admission records. The registrations are considered microscopically verified when the diagnosis is based on a malignant histological or cytological reports. The majority (98%) of the cases were microscopically verified. We excluded from the study patients for whom the proof of cancer could not be made or the medical file is incomplete or unexploitable. The borderline tumors and cases of intraepithelial neoplasia were also excluded from the study. Patients who do not reside in Eastern Morocco or for whom the place of residence was not specified were also excluded from the analysis.

We followed the registration rules, defined by the International Agency for Research on Cancer (IARC). The registered cases were coded according to the third edition of the International Classification of Diseases for Oncology (ICD-O-3) [17]. For tabulation of results, these were converted to the 10th revision of the ICD-O [18]. A form has been used for collecting information recorded on each case, such as the record number, name and surname of the patient, gender, age, family status, occupation, place of residence, health insurance, basis of diagnosis, tumor site and histology, incident date, and age at diagnosis. Gender has been indicated for all cases reported to the Hassan II Regional Oncology Center during the period of the study. However, age was not reported for 2.07% of men and 2.35% of women.

### Analysis

Data collection was performed on Excel. Statistical analysis was performed using SPSS software version 21.0. Crude incidence rates (CR) and Age-specific incidence rates (Ai) were calculated as previously described [19, 20]. The rates were expressed per 100,000 person per year [21]. Incidence cases which were registered during the period between January 2006 and December 2012 were used for the calculation of the incidence rates. National population censuses are conducted in Morocco every 10 years, and the HCP provides estimates of the growth rate of the Moroccan population for each year. In this paper, the census conducted in 2004 was used to elaborate the estimates of the population of Eastern Morocco during the period from 2006 to 2012. The annual percent change (APC) was calculated as previously described [21, 22] using the formula: APC = [exp (β) - 1] × 100, where β is the parameter estimate obtained on fitting period of event as a continuous variable to the logarithm of the rate. For the Chi-squared test, the results are considered significant when p (degree of significance) is less than 0.05, very significant when *p* < 0.01 and highly significant when *p* < 0.001.

## Results

### Cancer profiles in eastern Morocco

A total of 7872 incident cases of cancer were registered at the Hassan II Regional Oncology Center (ROC) since it was created in October 2005 until December 2012. Of these, 5220 cases were women and 2652 were men, with a female to male ratio of 1.97 (*p* < 0.001) (Table 1). The mean age at diagnosis was 58 years for males and 52 for females and 94% of the patients aged over 30 years.

**Table 1:** Female to male ratio of all incident cancer cases in Eastern Morocco as compared to other studies

Studies	Female cases	Male cases	F/H
*This study*	*5220*	*2652*	*1.97*
Morocco. Fez [25]	2622	2910	0.90
Morocco. Rabat [10]	1232	1241	0.99
Morocco. Casablanca [12]	6372	5551	1.14
Tunisia [22]	2474	3203	0.77
Egypt [24]	57425	57558	0.99
China [21]	18418	33515	0.54
France [23]	135895	183 485	0.74
European Union [1]	1206	1430	0.84
USA [1]	779	825	0.94

F/M sex ratio-female cases/male cases

The distribution of cancers in our study is shown in Table 2. Breast cancer came first, and exceeded by far the other cancers, with 30.75% of all cancers. The cervix uteri cancer came in the second position with 9.10%, followed respectively by colon-rectum cancer (8.16%), lung cancer (7.53%), nasopharyngeal cancer (5.03%), and stomach cancer with 4.43% (Table 2).

**Table 2:** Distribution of the 7,872 incident cases by cancer type, October 2005-December 2012

Type of cancer	Female	Male	Total	%
	N.	N.	N.	
All incident cases	5220	2652	7872	100
Breast	2368	53	2421	30.75
Cervix uteri	716	-	716	9.1
Colon-rectum	333	309	642	8.16
Lung	90	503	593	7.53
Nasopharynx	153	243	396	5.03
Stomach	164	185	349	4.43
Skin	109	145	254	3.23
Prostate	-	220	220	2.79
Ovary	197	-	197	2.5
Brain	77	94	171	2.17
Bladder and urinary tract	33	126	159	2.02
Bone	62	70	132	1.68
Thyroid	99	23	122	1.55
Esophagus	42	52	94	1.19
Pancreas	48	43	91	1.16
larynx	12	78	90	1.14
Gallbladder	67	12	79	1
Oral cavity and oropharynx	39	37	76	0.97
Corpus uteri	75	-	75	0.95
Soft parts	32	42	74	0.94
Liver	35	24	59	0.75
Kidney	19	24	43	0.55
Peritoneum	28	11	39	0.5
Vulva	32	-	32	0.41
Salivary gland	11	19	30	0.38
Eye	10	13	23	0.29
Testicles	-	20	20	0.25
Pharynx (unspecified)	8	9	17	0.22
Small intestine	10	4	14	0.18
Vagina	10	-	10	0.13
Tonsil	0	5	5	0.06
Mediastinum	1	3	4	0.05
Pleura	2	1	3	0.04
Trunk	1	-	1	0.01
Penis	-	1	1	0.01
Other	337	283	620	7.88

% percentage of the 7,872 incident cases; N number of incident cases

The gynecological and breast cancers (combined) are the most common tumors with 44.14% of all cancers, followed respectively by digestive cancers (16.9%), head and neck cancers (8.1%), and thoracic cancers (7.66%). Breast cancer occupies the first place in the gynecological and breast cancers with 69.66%, followed respectively by cervix uteri cancer (20.6%), ovarian cancer (5.65%) and corpus uteri cancer (2.15%). Regarding digestive cancers, colon-rectum cancer was in the first position with 48.34%, followed respectively by stomach cancer (26.28%), esophageal cancer (7.07%), pancreatic cancer (6.69%), cancer of the gallbladder (5.8%), liver cancer (4.44%) and cancer of the small bowel (1.02%).

**Fig. 1:**
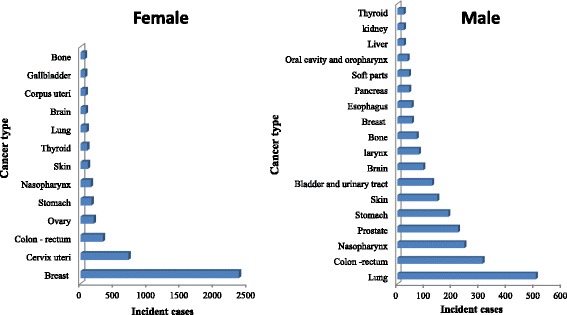
Distribution of incident cases by cancer type and by sex in Eastern Morocco

In women, breast cancer was almost half of female cancers with 45.36% of cases, and with a mean age at diagnosis of 48.7 years ±11.4. The cervix uteri came in the second position with 13.72%, followed respectively by colon-rectum cancer (6.38%), ovarian cancer (3.77%), and stomach cancer (3, 14%) (Fig. 1). In men, lung cancer was the most frequent malignancy with 19% of male cancers, followed respectively by colon-rectum cancer (11.65%), nasopharyngeal cancer (9.16%), prostate cancer (8.3%), and stomach cancer (7%) (Fig. 1). In our study, we found that men were more affected by lung cancer than women, with a male to female ratio of 5.6. The difference between sexes was highly significant (*p* < 0.001). Nasopharyngeal cancer affects both men and women but with a male to female ratio of 1.6 (*p* < 0.001), and the mean age for both sexes was 46 years.

### Trends in crude incidence rates in eastern Morocco

**Fig. 2:**
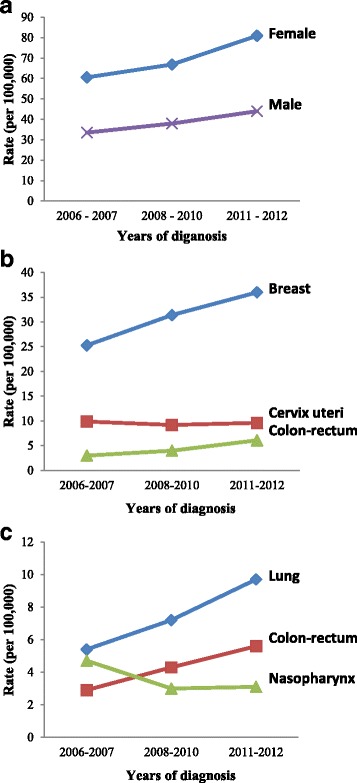
Trends in crude incidence rates in Eastern Morocco for: **a.** all cancers combined by sex. **b.** the top three leading sites in females. **c.** the top three leading sites in males

The crude incidence rates (CR) by sex of all cancers combined and of the major cancers were calculated for each year from 2006 to 2012 (Table 3) and the trends in CR were presented in three time periods: 2006–2007, 2008–2010 and 2011–2012 (Fig. 2). In women, the CR of all cancers combined increased throughout the study. The rates rose from 56.6 to 80.3 per 100,000 between 2006 and 2012 at an annual rate (APC) of 6% (Table 3, Fig. 2). The CR for breast cancer increased significantly during the period 2006 to 2012 at an APC of 8.1% (Table 3, Fig. 2). For colon-rectum and ovarian cancers, a significant increase was also observed at an APC of 18.7% and 9.9% respectively. However, the CR for cervix uteri cancer increased slightly by 1.6% during the study period, and that of the nasopharynx was decreasing (Table 3, Fig. 2).

**Table 3:** Crude incidence rates of the major cancers in Eastern Morocco, 2006-2012

Type of cancer	ICD-10		CR								APC
			2006	2007	2008	2009	2010	2011	2012		2006-2012
Female											
All cancers	C00-C90		56.6	64.5	68.5	61.3	70.9	81.6	80.3		+6%
Breast	C50		23	27.6	35.8	27	31.2	35.2	36.8		+8.1%
Cervix uteri	C53		9.2	10.6	11.2	7.4	8.9	9	10.1		+1.6%
Colon-rectum	C18-C20		2	3.9	3	4.2	4.9	6.5	5.6		+18.7%
Ovary	C56		1.7	2.9	2.4	2.5	2.7	3.3	3		+9.9%
Nasopharynx	C11		2.4	2.3	1.8	1.2	1.5	1.7	1.5		-7.3%
Male											
All cancers	C00-C90		32.3	34.8	35.7	36.3	42.0	45.5	42.6		+4.7%
Lung	C34		5.3	5.5	6.7	7.4	7.5	10.5	8.9		+9%
Colon-rectum	C18-C20		3.2	2.6	4.8	4	4	5.1	6		+11%
Nasopharynx	C11		5.1	4.3	2.8	3	3.3	3.6	2.6		-10.6%
Prostate	C61		3.1	2.6	2.5	1.8	4.3	3.7	4.3		+5.6%

CR crude incidence rate per 100,000; APC annual percent change

In men, the CR for all cancers combined increased throughout the study. The rates rose from 32.3 to 42.6 per 100,000 between 2006 and 2012 at an APC of 4.7% (Table 3). For lung, colon-rectum, and prostate cancers, there is a significant increase in CR between 2006 and 2012 by 9%, 11%, 5.6% and 20% respectively (Table 3, Fig. 2). However, CR for nasopharynx cancer significantly decreased between 2006 and 2012 by 10.6% (Table 3, Fig. 2).

### Age-specific incidence rates of nasopharynx cancer in eastern Morocco

**Fig. 3:**
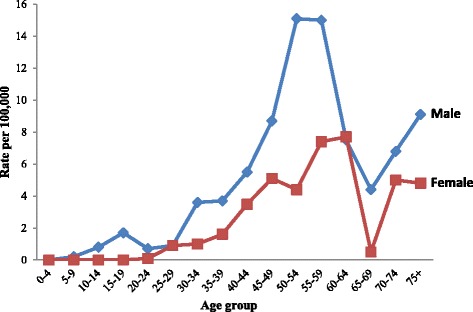
Age-specific incidence rates of nasopharynx cancer in Eastern Morocco

The age-specific incidence rate of nasopharynx cancer in male has shown a bimodal distribution with a first small peak in children (15–19 years), and a second large peak at the age group 50–59 years (Fig. 3). After this second peak, the incidence declined in the older age groups. The incidence rate showed a strong decrease at the age group 60–69, probably due to the age during admission being reported in round numbers in the older age groups. Indeed, an equivalent decrease was noticed at this age group for most cancers analyzed. In women, the incidence rate increased gradually with age until it reached a peak in the 55–64 age groups, and then declined in the older age groups. Several differences were observed between men and women: the incidence was higher in men than in women. The highest age-specific incidence rate was observed in a slightly later age group in female than in male (55–64 age groups versus 50–59 respectively). More importantly, the first peak observed in male children was absent in female children (Fig. 3).

### Socioeconomic profile of incident cancer cases in eastern Morocco

To improve the management of cancer patients and to plan and develop programs for cancer control and prevention in a region, it is very important to know its socio-economic context. Therefore, we have analyzed the profile of incident cancer cases registered at the Regional Oncology Center. As shown in Tables 4, 79% of cancer patients came from urban areas and 21% from rural areas (*p* < 0.001). The majority of the patients were unemployed (83% of cases), followed respectively by the following categories: retired (5%), self-employed (4.2%), employed (3.5%), farmer (1.8%), students (1.1%), and finally day laborer (0.9%) (*p* < 0.001) (Table 4). Moreover, the majority of patients did not have health insurance (85.3% of cases) (Table 4).

**Table 4:** Socio-demographic structure of the incident cancer cases in Eastern Morocco, October 2005-December 2012

	N.	%
Place of residence		
Urban	6,198	78.7
Rural	1,674	21.3
Employment status		
Employed	272	3.5
Self-employed	324	4.2
Unemployed	6,398	83.4
Retired	388	5.1
Farmer	140	1.8
Student	82	1.1
Day laborer	71	0.9
Health Insurance status		
Without health insurance	6,571	85.3
With health insurance	1,134	14.7

N number of incident cases with known information; %

## Discussion

Cancer is one of the major health problems worldwide [1, 4, 5]. Hence, it is very important to conduct epidemiological studies to identify risk factors and to develop programs for cancer control and prevention. In Western countries, the risk of cancer increases with age, and therefore, individuals aged 65 and over have the highest incidence rates [23]. In addition, because of early diagnosis and more effective treatments, there have been notable improvements in survival for most cancers [23]. Unlike Western countries, cancer seems to affect a younger population in our study. The most affected age group by cancer was 45–54 years, which accounted for 27% of cases, and the mean age of patients diagnosed with cancer was 54.1 years. It is important to mention that childhood cancer (0–14 years) accounted for only 0.5% of all cancers, and were under-represented in this study because the Regional Oncology Center typically manages adult cancer patients. Therefore, the age of occurrence of cancer in Eastern Morocco may be even younger. This finding is consistent with our recently published report on caner prevalence in Eastern Morocco [14].

In our study, the mean age of patients diagnosed with cancer was higher in men than in women (58.1 years versus 52.1 years). This indicates that women are affected by cancer at a younger age than men (*p* < 0.001). Similar results were observed with the Moroccan populations of Casablanca, Rabat and Fez [8, 12, 24, 25].

As shown in Table 1, the sex ratio observed in our study is much higher than that reported in Casablanca, Rabat and Fez, as well as in other countries in North Africa, Asia, Europe and USA [8, 9, 12, 23, 25–28]. Moreover, the crude incidence observed in 2012 was higher in females than males (80.3 per 100,000 women versus 42.6 per 100,000 men). Since the structure of the population of Eastern Morocco showed a sex ratio of female to male of 1.06 during the study period 2006–2012 [13], these data suggest that in Eastern Morocco, women are more affected by cancer than men, which is in agreement with our recently published report on caner prevalence in Eastern Morocco [14]. This great difference in incidence between men and women can be explained by differences in lifestyle, and/or the willingness to treat cancer between men and women.

Our retrospective analysis showed that for both sexes combined and for all cancer sites, breast cancer was the commonest followed by cervix uteri, colon-rectum, lung, nasopharynx, and stomach cancers. In males, lung cancer ranked first, followed respectively by colon-rectum, nasopharynx, prostate, and stomach cancers. Among the females, breast cancer was the most frequent, followed respectively by cervix uteri, colon-rectum, ovarian, and stomach cancer. These distribution patterns of cancers are quite different from those observed in Rabat and Casablanca registries [10, 11, 29]. For example, in females, the thyroid cancer was the third most common cancer in Rabat and Casablanca, while it was ranked at the eighth position in our study [10, 11]. Similarly, in men, prostate cancer was the second most common cancer in Rabat and Casablanca while it was ranked at the fourth position in our study [10, 11]. In this study, we found that incidence rates for most cancers are rising in Eastern Morocco. Indeed, the crude incidence rate for all cancers combined has increased from 56.6 to 80.3 per 100,000 per year in females and from 32.3 to 42.6 per 100,000 per year in males during the 2006–2012 period.

Breast cancer was the most common cancer in Eastern Morocco, with a mean age at diagnosis of 48.7 years ±11.4. This result is similar to that observed in the cancer registries of Casablanca (48.1–49.5 years ±11.3) [29, 30]. However, a different picture is observed in Western countries where breast cancer affects older women [31, 32]. The CR of breast cancer has increased from 2006 to 2012 in Eastern Morocco (23 to 36.8 per 100,000). This rising in trend may be explained by the breast cancer national screening program which has been adopted in Morocco for the period 2010–2019 in the context of the National Cancer Prevention and Control Plan [33]. However, there has been a significant increase in the trend before the program had been implemented (2006–2008), suggesting that this rising in trend for breast cancer is linked to one or more of the known risk factors for breast cancer. It is possible that some of the increase is related to declines in fertility, since the number of births has declined from 4.5 in 1987 to 2.2 in 2010 [34]. The rising in trend may also be related to later age marriage, since the mean age at marriage of women has steadily increased from 22.2 years in 1982 to 26.6 in 2010 [34]. Several other risk factors could be involved in the trend, like changes in diet, physical activity, lifestyle, genetic factors, age of menarche, exposure to hormones and breastfeeding.

Colon-rectum cancer was the second most common cancer in males and the third in females in Eastern Morocco. The CR of colon-rectum cancer has increased from 2006 to 2012 in Eastern Morocco in both sexes. This rising incidence trends for colon-rectum cancer may be linked to changes in dietary habits. Indeed, it has been well established that a high calorie diet and rich in animal fats, mostly absorbed in the form of red meat, and with few vegetables and fiber, is associated with an increased risk of colon-rectum cancer. Alcohol and smoking also increase the risk for colon-rectum cancer [35–37]. Conversely, a diet providing little fat, lots of vegetables and possibly rich in fiber, has a protective effect [38–42]. In Eastern Morocco, there is still no screening program for colon-rectum cancer. Therefore, the observed rising incidence trend in this cancer in both males and females justifies the need to establish programs for colon-rectum cancer control and prevention in Eastern Morocco.

Lung cancer was the most common cancer in males in Eastern Morocco. The incidence of Lung cancer in males has increased from 2006 to 2012 in Eastern Morocco (5.3 to 8.9 per 100,000). Lung cancer is the leading cancer in males in developing countries. However, it ranks second after prostate cancer in Europe [43]. This is also the leading cause of cancer death [44]. The association between lung cancer and smoking is well established [45–47]. Therefore, the observed rising incidence trend in lung cancer in Eastern Morocco may be linked to the increase in the prevalence of smoking [48, 49]. Thus, it is important to develop tobacco control measures as soon as possible to avoid future increases in lung cancer in Eastern Morocco.

Nasopharynx cancer was the third most common cancer in males and the sixth among women in Eastern Morocco. In both sexes, nasopharynx cancer has shown a steady decline in incidence throughout the study period. This decreasing trend can be linked to changes in diet, lifestyle or exposure to toxic smoke. Indeed, the association between nasopharynx cancer and smoking, working in conditions with poor ventilation, infection with Epstein Barr virus, and the rich traditional food preservatives has been demonstrated [50–53]. In males, the age-specific incidence rate of nasopharynx has shown a bimodal distribution with the first peak in children (15–19 years), and the second peak in adults (age group 50–59 years). This result is similar to that observed in the cancer registry of Setif in Algeria (two peaks at 15–24 and 50–59 years) [54] . However, a different picture is observed in the cancer registry of Casablanca, where the first peak was observed earlier (10–14 years age group) [11, 22, 54]. This difference may be explained by the under-representation of childhood cancer in this study because the ROC typically manages adult cancer patients. In females, the incidence rate increased gradually with age and reached a peak in the 55–64 years age group. Such a finding is different from the one reported in Casablanca, where the age-specific incidence rate showed that the incidence rate reached a peak in adults at an earlier age group (40-44 years) [11, 22, 54]. These differences may be explained by a lower prevalence of smoking among females in Eastern Morocco compared to Casablanca. Further studies are needed to determine the causes of these differences.

Cancers are caused by several factors, including genetic, geographical, socioeconomic, and cultural factors [55–57]. Thus, the epidemiological characteristics of cancer vary from one region to another, and even within the same country. In Tunisia, for example, cancer registries of Sousse, Sfax and northern Tunisia have shown quite different cancer incidence rates [22]. Similarly, the data presented here on cancer patterns in Eastern Morocco are different from those of the Casablanca and Rabat cancer registries [10, 29]. The low socioeconomic status could in part explain the general increase in the incidence of cancer of most major sites in Eastern Morocco during the study period. Indeed, 79% of incident cancer cases registered at the Regional Oncology Center were from urban areas and 21% from rural areas (*p* < 0.001). Moreover, 83% of patients were unemployed and 85% have no health insurance. It is worth noting that according to the High Commission for Planning, the employment rate for women in eastern Morocco is only 11.1%, compared to 65.7% for men [58]. Therefore, men may be at lower risk due to engaging in occupations requiring greater physical activity. Although occupational differences may explain some of the sex difference, other factors such as diet and hormonal differences must be considered.

## Conclusion

In the absence of a cancer registry in Eastern Morocco, it is difficult to understand the epidemiology of this disease and develop programs for cancer control and prevention. Since Hassan II Regional Oncology Center was the only specialized cancer hospital for cancer management in Eastern Morocco during the study period, these results are of great value and provide a valuable tool for cancer control and prevention in Eastern Morocco. Our results confirm the gravity of this problem, and provide epidemiological knowledge about cancer which is necessary for health care planning. This work is of great interest to the health system since any prevention policy cannot be implemented without epidemiological studies and very thorough statistical analysis. Indeed, the rates of many cancers are rising and require the development of effective programs for cancer control and prevention. A better and easier access to cancer care is a priority, given that the majority of patients who are treated in the Regional Oncology Center are unemployed, and do not have medical care coverage. It is also essential to initiate the establishment of a regional cancer registry to allow the surveillance of cancer trends and to plan a program for cancer control and prevention in Eastern Morocco.

## Abbreviations

APC: Annual percent change
CR: crude incidence rates
HCP: High Commission for Planning
HM: hematological malignancies.
IARC: International Agency for Research on Cancer
ICD-O: International classification of diseases for Oncology
ROC: Hassan II Regional Oncology Center
WHO: World health organization
